# Can Resveratrol Treatment Control the Progression of Induced Periodontal Disease? A Systematic Review and Meta-Analysis of Preclinical Studies

**DOI:** 10.3390/nu11050953

**Published:** 2019-04-26

**Authors:** Eric Francelino Andrade, Débora Ribeiro Orlando, Amanda Melo Sant’Anna Araújo, James Newton Bizetto Meira de Andrade, Diana Vilela Azzi, Renato Ribeiro de Lima, Adalfredo Rocha Lobo-Júnior, Luciano José Pereira

**Affiliations:** 1 Institute of Agricultural Sciences, Universidade dos Vales do Jequitinhonha e Mucuri—UFVJM, Rua Vereador João Narciso, n.º 1380–Bairro Cachoeira, Unaí, Minas Gerais 38610-000, Brazil; ericfrancelinoandrade@gmail.com (E.F.A.); deboraribeiro.orlando@gmail.com (D.R.O.); amanda.araujo@ufvjm.edu.br (A.M.S.A.); james.andrade@ufvjm.edu.br (J.N.B.M.d.A.); rochalobo@gmail.com (A.R.L.-J.); 2 Department of Veterinary Medicine, Universidade Federal de Lavras—UFLA, Mail Box 3037, Lavras, Minas Gerais 37200-000, Brazil; dianavilelaazzi@hotmail.com; 3 Department of Exact Sciences, Universidade Federal de Lavras—UFLA, Mail Box 3037, Lavras, Minas Gerais 37200-000, Brazil; rrlima@ufla.br; 4 Department of Health Sciences, Universidade Federal de Lavras—UFLA, Mail Box 3037, Lavras, Minas Gerais 37200-000, Brazil

**Keywords:** 2,3,5,4′-tetrahydroxystilbene-2-O-β-glucoside, Periodontitis, Oral Health, Mouth Diseases, Functional Food

## Abstract

Resveratrol is an anti-inflammatory compound found in several foods. Periodontal disease (PD) is associated to other systemic diseases, and inflammation may be responsible for the association. Consequently, controlling inflammation not only may benefit oral health but also may assist with the management of other chronic inflammatory conditions. We aimed to investigate the effects of resveratrol administration on PD control in preclinical studies. A systematic search was performed for scientific articles using both electronic databases and a manual search using combinations of the following keywords: “resveratrol” OR “3,5,4′-trihydroxystilbene” AND “periodontal disease” OR “periodontitis” OR “gingivitis”. Only in vivo original studies investigating resveratrol treatment on experimental animal models of PD were selected. A quality assessment of the studies was performed using the Animal Research Reporting In Vivo Experiment (ARRIVE) guidelines, and the risk of bias was assessed using the Syrcle tool. The search returned 570 articles, and 11 matched the inclusion criteria. A meta-analysis showed that resveratrol treatment attenuated alveolar bone loss (τ^2^ = 0.0041; 95% CI: −0.14; −0.04). The ARRIVE criteria reported a good quality of studies in general (mean score 28.5 ± 2.5). However, five Syrcle domains indicated a high risk of bias or did not present information clearly. We concluded that, in preclinical studies, resveratrol treatment prevented PD progression.

## 1. Introduction

Resveratrol (3,5,4′-trihydroxystilbene) is a polyphenol stilbene found in red wine [1], peanuts [2], apples, several vegetables and berries, and others [3]. This compound has gained attention due to the “French Paradox” that indicates a low incidence of cardiovascular diseases and even consuming a high saturated fat diet [4]. The explanation for this paradox is associated with the consumption of red wine in France that naturally contains “therapeutic doses” of resveratrol [5]. Recently, resveratrol was also linked to several other health benefits such as cardioprotective effects, antitumor activity, and a life span increase [6,7]. Consequently, resveratrol has been investigated for the prophylaxis and therapeutic treatment of periodontal disease (PD) based on its anti-inflammatory and antioxidant properties [3,8]. 

PD embraces a group of inflammatory conditions affecting teeth supporting tissues [9]. It may be limited to the gums (gingivitis) or may affect the alveolar bone and periodontal ligament (periodontitis), causing apical epithelial migration and alveolar bone resorption [9]. This inflammation frequently involves a host response against biofilm accumulation and is mostly clinically asymptomatic in its early stages [10]. The direct consequences of PD involve gingival bleeding, tooth mobility, and even tooth loss, leading to aesthetic impairment and a poor quality of life [10]. In addition, PD is also associated with other systemic diseases such as cardiac, renal, and respiratory dysfunction; rheumatoid arthritis; metabolic syndrome; and even cancer [11].

It is estimated that the prevalence of PD in the world population exceeds 50% of adults older than 30 year. Approximately 10% of these individuals present the more severe forms [12,13]. Population growth and aging have been contributing to an increase of severe periodontitis cases [13,14], generating a universal public health issue [13,15]. Risk factors for the development and progression of PD involve mainly genetic polymorphisms, stress, obesity and diabetes, smoking, and alcoholism [16]. Proper oral hygiene and regular dental care (including scaling and root planning) are conventional therapies employed in dental practice [17]. Alternatively, maintaining an ideal weight and consuming a high-quality diet can also help to improve periodontal health [18], besides avoiding smoking and excessive alcohol drinking. 

Animal studies have evaluated the effects of resveratrol administration on experimentally induced periodontitis showing promising results [19,20,21]. However, the issue is not yet fully understood, making difficult the incorporation of resveratrol as a therapeutic/preventive agent clinically. Clinical studies investigating these parameters in humans are scarce, maybe because there is still no evidence from preclinical studies to support its incorporation into an improvement to disease prevention/treatment. In this regard, a systematic review with a meta-analysis can bring light to this question [22]. The objective of this study was to investigate the effects of resveratrol administration on the periodontal status in preclinical animal studies.

## 2. Materials and Methods

### 2.1. Focused Question

The Participants, Interventions, Control, and Outcomes (PICO) format was used to formulate the focused question “Can resveratrol administration control the progression of induced periodontal disease?” 

In this sense, the PICO represent the following: P: laboratory animals with induced periodontal disease (all species); I: resveratrol administration; C: no resveratrol or vehicle only; and O: alveolar bone loss and an expression of cytokines.

To ensure the quality of this study, we adopted the criteria of The Preferred Reporting Items for Systematic Reviews and Meta-Analyses (PRISMA) [23] and the protocol for the preparation, registration, and publication of systematic reviews of animal intervention studies described by Vries et al. [24]. 

### 2.2. Eligibility Criteria

#### 2.2.1. Type of Studies

Only preclinical (animal models) studies using resveratrol administration on an induced periodontal disease were eligible for this review. There was no restriction of the publication date or language. 

#### 2.2.2. Research Strategy

An electronic search in five databases were performed in February 2019: PubMed (http://www.ncbi.nlm.nih.gov), ScienceDirect (https://www.sciencedirect.com), Scielo (http://www.scielo.org/php/index.php), Scopus (https://www.scopus.com), and Web of Science- ISI Web of Knowledge databases (https://login.webofknowledge.com). The following search strategy was used: “resveratrol”, “3,5,4′-trihydroxystilbene” AND “periodontal disease”, “periodontitis”, OR “gingivitis”. Additionally, in order to avoid that any relevant article was not selected, we performed an additional search using the keywords such as “melinjo” and “red wine” which also combined with the terms “periodontitis” and “periodontal disease”. A manual search was conducted in the reference lists of the included studies. A manual search was also conducted in the reference list of all retrieved articles.

### 2.3. Screening Methods and Selection of Studies

We selected only original in vivo studies that investigated the effect of resveratrol administration on an induced periodontitis progression in animal models. There was no restriction regarding the publication language, resveratrol administration, PD induction protocol, trial period, genus, species or sample size, and/or publication date. The exclusion criteria were literature reviews, letters to the editor, editorials, in vitro studies, book chapters, conference abstracts, and drug development without an in vivo analysis. 

Two independent reviewers were trained and conducted searches in accordance with inclusion/exclusion criteria. Articles were selected based on abstracts and/or title. In cases of a nonconformity among the selected items, a third reviewer was called for consensus. After a full text analysis, a new discussion was performed in order to determine the possible extra exclusion of studies.

Bone loss data for the treated and non-treated (control) groups were recorded in order to calculate the relative reduction due to treatment. Absolute values were annotated from original tables or estimated from figures in the retrieved articles.

### 2.4. Quality Criteria Assessment

We performed a quality criteria evaluation of the selected studies following the Animal Research Reporting In Vivo Experiment (ARRIVE) guidelines. It contains a predefined grading for 20 categories [25,26]. Each criterion received a score as reported previously [25,26,27,28]. Items “1”, “4”, “11”, and “14” received a minimum score of 0 and a maximum score of 1 (0 = inaccurate, not concise, or not reported; 1 = accurate, concise, or reported). The other items (2, 3, 5, 6, 7, 8, 9, 10, 12, 13, 15, 16, 17, 18, 19, and 20) received a minimum score of 0 and a maximum score of 2 (0 = clearly inaccurate or not reported; 1 = possibly accurate, unclear, or incomplete; 2 = clearly accurate). The sum of the scores varied from zero to 36 points. The maximum score by columns were also calculated to obtain the quality score sum by category, as described by Javed et al. [27]. Thus, the maximum score found by category was the maximum possible score. We calculated a ratio Quality Score/Maximum Score, generating three possible range coefficients in which 0.8–1 was considered “excellent”, 0.5–0.8 was considered “average”, and scores below 0.5 were considered “poor” [27].

### 2.5. Bias Risk Assessment

We evaluated the risk of bias using the Systematic Review Centre for Laboratory Animal Experimentation (SYRCLE) tool [29]. This tool contains 10 entries related to the selection, performance, detection, attrition, and reporting bias among others. The classification of the quality criteria and risk of bias were performed by two independent authors. Any disagreement was resolved by consensus with a third reviewer.

### 2.6. Statistical Analysis

A meta-analysis was performed by using the META package [30] of the R statistical software [31]. In order to avoid a methodological heterogeneity in meta-analysis, only studies that evaluated a bone loss through a measurement of the cementoenamel distance to the bone crest in methylene blue stain specimens were included in forest plot. The inverse variance method was applied and the DerSimonian–Laird method was used to estimate the difference between-studies variance (τ^2^). The mean difference was used as effect measure, i.e., the mean value in resveratrol group minus the mean value in control group (without resveratrol).

A random effect model was used for the meta-analysis. The summary of the effect measure was depicted in a forest plot. The mean difference (MD) and 95% confidence intervals (CI) were presented in the plot. In this design, for each study, the mean value, standard deviation, and sample size were reported for both experimental (with resveratrol) and control (without resveratrol) groups.

The publication bias was not quantitatively evaluated by Egger test or funnel plot despite the number of studies grouped in the funnel plot [32]. 

## 3. Results

**Figure 1:**
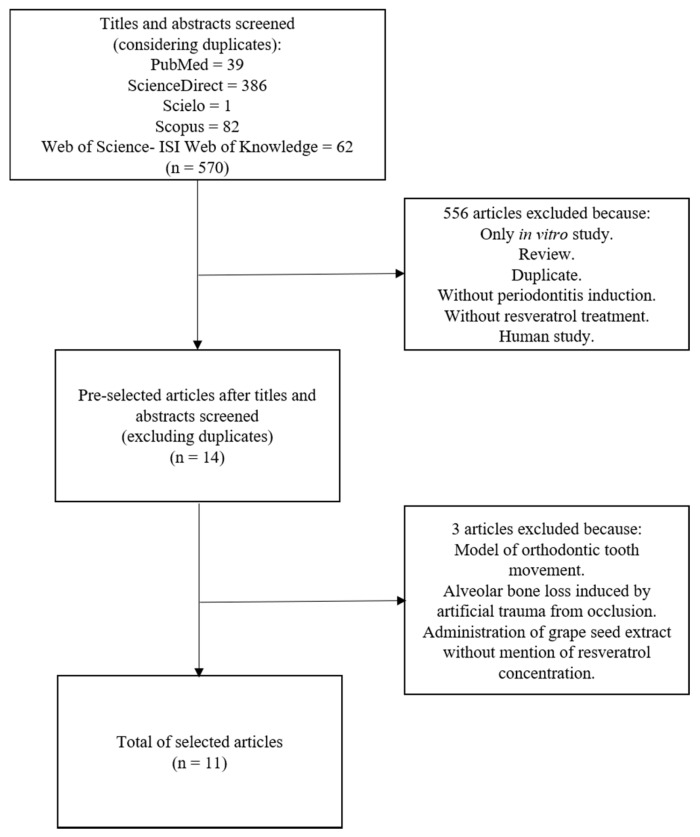
A flowchart of the studies selection.

The initial searches in all databases returned 570 articles, and 14 of these were preselected. After full text analysis, 11 papers met the inclusion criteria for this review (Figure 1).

Two of the preselected articles were excluded despite an evaluation of the periodontal tissue during orthodontic tooth movement [33] and an alveolar bone loss induction by artificial occlusal trauma [34]. A third study was excluded because the concentration of resveratrol in a grape seed extract was not informed [35].

Additionally, one study evaluated the effects of resveratrol administration on periodontitis in humans [36]. In this study, four weeks of daily intake of capsules containing 480 mg of resveratrol decreased the mean pocket depth in diabetic patients with chronic periodontitis compared to patients consuming a placebo. 

Out of the 11 selected studies, seven used Wistar rats as experimental model [20,37,38,39,40,41,42]. Another two studies used Sprague–Dawley rats [21,43]. Two additional papers used C57BLKS/J-db/db [19] and C57BL/6J wild-type mice [44]. All studies reported using males, and the age at the beginning of the experiment protocol ranged from 8–10 weeks for rats and from 6–8 weeks for mice. The duration of induced PD ranged from 8 [43] to 30 days [41,42]. All articles reported the ligature protocol. However, Bhattarai et al. [21] and Zhen et al. [19] also administered lipopolysaccharide (LPS) or a medium containing *Porphyromonas gingivalis*, respectively. 

Resveratrol administration routes included intraperitoneal [44], subcutaneous [21], oral [38], or via gavage [19,20,37,39,40,41,42,43]. Doses varied from 10 mg/kg [20,21,37,38,39,40,41,42] to 25 mg/kg [43]. The duration of treatment also varied from seven [43] to 30 days [20,39,40,41,42]. Ikeda et al. [44] used a single dose corresponding to 0.001% (*w*/*w*) of the body weight.

Alveolar bone loss (ABL) was evaluated in 10 of the 11 selected studies, and a treatment with resveratrol attenuated this parameter in nine of these articles [19,20,21,37,38,40,41,42,44]. In six studies, ABL was evaluated through morphometry using a cement–enamel junction distance to the bone crest in methylene blue stained samples [19,20,37,40,41,42]. One study also evaluated ABL through a morphometry of the cement–enamel junction distance in Hematoxylin and Eosin (H&E) stained specimens [21]. Three studies evaluated ABL using Micro-computed tomography (micro-CT) [21,38,44], and one study used radiographic examination [36]. Oxidative stress and inflammatory improvement were also reported for animals receiving resveratrol [19,20,21,37,38,40,41,42,44]. Cirano et al. [32], on the other hand, found no differences for *Aggregatibacter Actinomycetemcomitans*, *Porphyromonas gingivalis*, and *Tannerella forsythia* concentrations in ligatures extracted from rats after resveratrol administration. 

Four studies evaluated the effects of resveratrol on PD associated with other conditions such as arthritis [42], smoke inhalation [20,40], and diabetes [19]. All articles indicated an improvement of PD after resveratrol administration. In one study [41], resveratrol was combined with curcumin. No synergism was observed for ABL or gingival IFN-γ. The main characteristics of the selected studies are described in Table 1 and Table 2. An average bone loss reduction with resveratrol ranged from 7.09% [38] to 61.52% [21] (Table 2).

### 3.1. Risk of Bias in Studies

The results of SYRCLE showed that 63% of studies were classified as low risk in the allocation details domain. In the domains “baseline characteristic” and “other bias”, 81% of the studies presented a low risk of bias. A high risk of bias was observed in all papers for the item “allocation concealment”, since no animal allocation was mentioned. The domains “random housing of animals” and “random selection for outcome assessment” showed an unclear risk of bias, 81% and 45%, respectively. Several studies did not provide enough information on the “blinding of participants” and were classified as unclear (36%) or as high risk of bias (63%), while only 0.9% of studies presented a low risk of bias for this item. Almost half (45%) of the studies scored as low risk of bias in both the domains “blinding of outcome assessment” and “incomplete outcome data” while the other half were scored as an unclear risk of bias because of lack of detailed information. Most of the studies (91%) presented a low risk of bias in the item “selective outcome reporting” (Table 3).

### 3.2. Quality Assessment of Selected Publications

The total quality score among the studies ranged from 18 to 32 (mean score 28.5 ± 2.5) out of a maximum of 36 points (Table 4) according to the ARRIVE guidelines. Eleven categories scored as “excellent”, while nine categories were classified as “average”. No category was classified as “poor”.

### 3.3. Meta-Analysis Results

**Figure 2:**
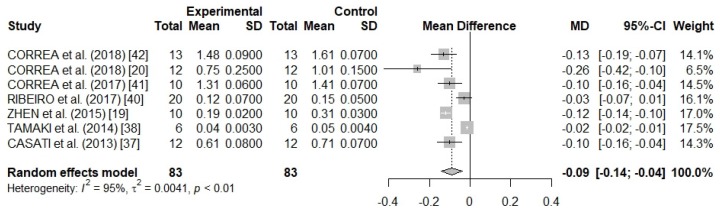
A forest plot of the meta-analysis for seven preclinical studies evaluating alveolar bone loss (cementoenamel junction distance to the bone crest) in methylene blue stain rat samples.

There was a high heterogeneity between the studies (*I*^2^ = 95%; *p* < 0.01), and for this reason, a random effects model was used in meta-analysis (Figure 2), since the subgroup meta-analyses and meta-regression must not be conducted due to few studies. Using the random effects model, it was possible to observe that the small studies received from 6.5 to 17.5% of the weights as well as that the pooled weighted mean difference was of −0.09 (τ^2^ = 0.0041; *p* < 0.01), which may be in a 95% CI from −0.14 to −0.04. This result indicates a significant effect in the reduction of the alveolar bone loss with the use of resveratrol.

## 4. Discussion

In this systematic review and meta-analysis, we investigated the effects of resveratrol administration on PD progression in preclinical studies. Out of the eleven selected studies, ten evaluated ABL and nine observed improvement of this parameter after resveratrol administration [19,20,21,37,38,40,41,42,44]. Such results may be related to an improvement of oxidative stress and anti-inflammatory properties [3,8]. 

Resveratrol increases the activity of antioxidant enzymes such as superoxide dismutase (SOD), catalase (CAT), and peroxidase (POD), which are key components against reactive oxygen species (ROS) [45]. Moreover, the host response to periodontal pathogens promotes local and systemic elevations of proinflammatory cytokines that alter the expression of the receptor activator of nuclear factor-kappa B ligand (RANKL) on the osteoblast surface [46]. RANKL is responsible for activating osteoclasts through its interaction with the receptor activator of nuclear factor-kappa B (RANK), initiating bone resorption [46]. High levels of ROS acting as intracellular signal transducers result in autophagy, which plays a dual role in periodontitis by promoting cell death or blocking apoptosis in infected cells [47]. In addition, ROS may influence the activation of signaling nuclear factor-κB (NF-κB), resulting in an increase of proinflammatory cytokines and, consequently, stimulating the differentiation of osteoclasts [47]. Considering that PD can be worsened due to the increase of pro-inflammatory cytokines and ROS, resveratrol acts on both fronts attenuating the progression of this disease [8]. 

One of the retrieved studies, Chin et al. [43] found no significant improvement in ABL in animals treated with resveratrol (even with the largest dose of 25 mg/kg/day). However, the treatment period was maintained for only seven days. [43]. It is possible that there is some mechanism related to the time of action or even to the administration period of this (prior to or following induction of PD). More studies should be conducted to determine the effective optimal dose and duration needed to attenuate ABL. Regarding toxicity, no side effects were reported for resveratrol consumption in any of the selected studies.

Regarding administration route, only two studies evaluated the injectable administration of resveratrol [21,44]. Even a single 0.001% (*w*/*w*) dose delivered one day prior to PD induction promoted ABL reduction, inflammatory profile, and oxidative stress improvements in C57BL/6J wild-type mice [44]. The highest percentages of ABL reduction were observed in studies where resveratrol was injected [21,44]. This fact may be related to the reduction of resveratrol bioavailability after gastrointestinal absorption [48].

The mechanisms involved in resveratrol regulation in periodontal inflammation have not yet been fully elucidated [21]. It is known that resveratrol can decrease the expression of toll-like receptor type 4 (TRL4) which is activated by lipopolysaccharides (LPS) [21]. An activation of TLR4 observed in chronic periodontitis increases the production of proinflammatory cytokines [49]. Another possible mechanism is by increasing anti-inflammatory mediators such as IL-4 [42], as well as a suppression of both matrix metalloproteinase (MMP-2 and MMP-9) and cyclooxygenase-2 (COX-2) [21]. IL-4 suppress the production of IL-17 and IL-1β [42], which play an important role in the periodontitis pathogenesis and inhibit both Th1 pro-inflammatory response and bone resorption [50]. 

All analyzed studies used rat or mice. Rodent periodontium has a good similarity with humans [51]. The induction of periodontitis involved the ligature in all experiments. This model has several advantages such as low cost and the possibility of investigation in a wide genetic variety of rodents, besides allowing the exploration of the interaction between the oral microorganisms and host response during the development of periodontitis [7]. Even though rats are not natural hosts for some bacteria found in human oral cavity, both *A. actinomycetemcomitans* and *P. gingivalis* have been reported in rodents’ microbiota after ligature [39,52,53]. 

Regarding the quality assessment of studies, most of the categories were classified as excellent or average. A previous systematic review [54] using the ARRIVE guidelines reported that most animal studies lack a clear indication of the reasons for choosing a particular animal model, contributing to a lower score for this item. This item also received the lowest score. This aspect is considered an important criterion for preclinical animal trials [54]. Thus, even if it is already well-known that some results of studies in rodents can be extrapolated to humans, it is important to highlight the relevance of the model used.

Another ARRIVE item that scored as low in several studies was the “interpretation/scientific implications” since only a few articles commented about the limitations and bias as well as mentioned the 3Rs (replacement, refinement, and reduction) principles adopted in experiments with animal studies. Similar results were also reported by Dereka et al. [55], in which only 10% of the studies attended this question. 

Among the risk of bias assessed, the domains “allocation concealment”, “random housing”, “blinding of participants and personnel”, “random outcome assessment”, and “blinding of outcome assessment domains” presented a high risk of bias or were not clearly described. These same domains were classified as unclear or with a high risk of bias according to the Syrcle tool in previous study [56]. In a systematic review of animal studies [54], 40% and 60% of studies presented a high risk of bias and an unclear risk in the “allocation concealment” domain, respectively. Randomized housing during the experiment reduces the bias risk once these conditions (such as lighting, humidity, temperature, etc.) are known to influence study outcomes [29]. An implementation of a blind evaluation in animal studies is also crucial, especially for subjective measurements [57]. 

This systematic review was conducted according to the PRISMA criteria [23] and the protocol for the preparation, registration, and publication of systematic reviews of animal intervention studies [24]. In order to minimize bias, each step of searching or ranking was performed by two independent researchers. Additionally, to prevent the exclusion of any article, a careful search was conducted. It is important to emphasize that, in the searches performed, we have not found previous systematic review or meta-analysis studies involving the main question addressed in this study. 

The results of meta-analysis demonstrated that, in rats, bone loss was significantly lower due to resveratrol administration. However, only a few studies were eligible for the meta-analysis (seven), and it was not possible to assess the publication bias. Considering the potential benefits of resveratrol on periodontal health, further studies should be conducted investigating the effects of this compound in PD models. In addition, future studies should focus on reducing the risk of bias, especially in the areas related to “allocation concealment” and "blinding of participants and personnel" which contributed to the greatest risk of bias. 

## 5. Conclusions 

We concluded from preclinical studies that resveratrol can improve periodontal disease probably due to the modulation of both oxidative stress and inflammatory profile. The results obtained through ARRIVE showed a good quality of the studies overall. However, the analysis by the Syrcle tool demonstrated that some aspects related to randomization and blinding should be considered to reduce the risk of bias.

The findings of this systematic review and meta-analysis demonstrated promising effects of resveratrol on periodontal disease, which may stimulate future studies in humans. Because of species-specific variables such as oral microbiota, dose-response effect, and the route of administration, the results in humans may vary from those observed in animals. Clinical studies are essential to confirm the results observed in animal studies.

## Figures and Tables

**Table 1:** The main features of the selected preclinical studies.

Author and Year of Publication	Animal Model (Age at Beginning of Study)	Groups (n/Group)	Characteristics of Periodontitis Induction Protocol	Time of PD Induction	Ligature Permanence	Route of Resveratrol Administration	Resveratrol Dose	Main Outcomes
CORRÊA et al. (2018) [42]	Male Wistar rats (NC)	1: PD + smoke inhalation + placebo (13) 2: PD + smoke inhalation + resveratrol (13)	Cotton ligature around both mandibular first and second maxillar molars.	26 days after beginning experiment.	11 days	Gavage	10 mg/kg of body weight 30 days (entire experiment).	Resveratrol reduced both ABL and gingival NADPH oxidase as well increased tissue levels of SIRT1 and SOD1.
CORRÊA et al. (2018) [20]	Male Wistar rats (NC)	1: PD + experimental arthritis + placebo (12). 2: PD + experimental arthritis + ibuprofen (12). 3: PD + experimental arthritis + resveratrol (12).	Cotton ligature around mandibular first molars.	At beginning of experiment	30 days	Gavage	10 mg/kg of body weight during 30 days (entire experiment).	Resveratrol treatment decreased ABL and increased serum IL-4 levels.
IKEDA et al. (2018) [44]	Male C57BL/6J wild-type mice (6–7 weeks old)	1: PD + placebo (NC) 2: PD treatment + placebo (NC) 3: PD + resveratrol (NC) 4: PD treatment + resveratrol (NC)	Silk suture around the gingival sulcus of the maxillar second molar. In PD treatment group ligature was removed 15 days before finishing the experiment.	At beginning of experiment	15 days in PD treatment group. In the other groups ligature remained 17, 20, and 22 days.	Intraperitoneal	Single dose 0.001% (*w*/*w*) of body weight on day 14.	Resveratrol decreased ABL, IL-1β, and oxidative stress Production of osteoclasts was inhibited by resveratrol.
CORRÊA et al. (2017) [41]	Male Wistar Rats (10 weeks old)	1: PD + placebo (10). 2: PD + resveratrol (10). 3: PD + curcumin (10). 4: PD + resveratrol + curcumin (10).	Cotton ligature around mandibular first molars.	At beginning of experiment	30 days	Gavage	10 mg/kg of body weight during 30 days (entire experiment).	Resveratrol decreased ABL and gingival IFN-γ. Resveratrol increased gingival IL-4.
RIBEIRO et al. (2017) [40]	Male Wistar rats (10 weeks old)	1: Control + placebo (20) 2: PD + smoke inhalation + placebo (20) 3: PD + smoke inhalation + resveratrol (20)	Cotton ligature around both first mandibular molar and second maxillar molar.	19 days after beginning experiment	11 days	Gavage	10 mg/kg of body weight during 30 days (entire experiment).	Resveratrol reduced linear ABL and increased interradicular bone density. It also reduced expression of RANKL and Th17/Th2 levels whereas increased serum levels of IL-4.
BHATTARAI et al. (2016) [21]	Male Sprague–DawleyRats (NC)	1: Sham (5) 2: Control + DMSO (5) 3: PD + DMSO (5) 4: PD + Resveratrol (5)	Elastic ligature between first and second maxillary molars and received 20 µl of 1 mg/mL LPS three times/week into the palatal gingivae	At beginning of experiment	14 days	Subcutaneous	5 mg/kg of body weight during 14 days (entire experiment).	Resveratrol attenuated ABL soft tissue damage and inhibited osteoclast formation. It also reduced COX-2, MMP-2, and MMP-9 levels. Resveratrol increased bone mineral density and SOD activity.
CHIN et al. (2016) [43]	Male Sprague–Dawley rats (8 weeks old)	1: control (10) 2: PD (10) 3: PD + 0.1 mg/kg/day of THSG (5) 4: PD + 10 mg/kg/day of THSG (5) 5: PD + resveratrol (5) 6: PD + 12.5 mg/kg/day of *P. multiflora* ethanol extracts (5) 7: PD + 25 mg/kg/day of *P. multiflora* ethanol extracts (5) 8: PD + 50 mg/kg/d of *P. multiflora* ethanol extracts (5)	Silk sutures around mandibular first molars	At beginning of experiment	8 days	Gavage	25mg/kg of body weight during 7 days.	Resveratrol treatment did not alter significantly (*p* = 0.054) periodontal bone-supporting ratio.
CIRANO et al. (2016) [39]	Male Wistar rats (10 weeks old)	1: Control (PD) + placebo (12) 2: PD + resveratrol (12)	Cotton ligature around first mandibular molar.	19 days after beginning experiment	11 days	Gavage	10 mg/kg of body weight during 30 days (entire experiment).	Resveratrol treatment did not alter concentrations of *Aggregatibacter Actinomycetemcomitans*, *Porphyromonas gingivalis*, and *Tannerella forsythia* in rats’ ligatures.
ZHEN et al. (2015) [19]	C57BLKS/J-db/db male mice (6–8 weeks old)	1: Untreated control (10) 2: PD + placebo (10) 3: PD + resveratrol (10)	Cotton ligature presoaked in a medium containing *Porphyromonas gingivalis* (108/mL) around maxillar first molars.	At beginning of experiment	28 days	Gavage	20 mg/kg of body weight during 28 days (entire experiment).	Resveratrol decreased ABL and decreased IL-1β, IL-6, IL-8, and TNF-α levels. The expression downstream signaling activation of TLR4 was attenuated.
TAMAKI et al. (2014) [38]	Male Wistar Rats (8 weeks old)	1: Control + water (6) 2: PD + water (6) 3: PD + resveratrol (6)	Ligature of thread placed around the right second molar of maxilla.	At beginning of experiment	20 days	Oral	10 mg/kg of body weight of melinjo resveratrol during 20 days (entire experiment).	Resveratrol ABL and activated the Sirt1/AMPK and the Nrf2/antioxidant defense pathways in inflamed gingival tissues. Resveratrol inhibits the NF-κB/MAPK pathway and lowered both serum IL-6 and TNF-α.
CASATI et al. (2013) [37]	Male Wistar Rats (10 weeks old)	1: PD + placebo (12) 2: PD + resveratrol (12)	Cotton ligature around mandibular first molar.	19 days after beginning experiment	11 days	Gavage	10 mg/Kg of body weight during 30 days (entire experiment).	Lower ABL and lower levels of IL-1β and IL-17 in resveratrol treated group.

Abbreviations: NC: Not clear. ABL: Alveolar bone loss. DMSO: Dimethylsulfoxide. MMP: Matrix metalloproteinases. IL: Interleukin. TNF-α: Tumor necrosis factor-α. COX-2: Cyclooxygenase-2. SOD: Superoxide dismutase: NADPH: nicotinamide adenine dinucleotide phosphatase oxidase. SIRT1: Sirtuin 1. IFN-γ: Interferon gamma. RANKL: Receptor activator of nuclear factor kappa-Β ligand. TRL: Toll-like receptor.

**Table 2:** A reduction of the bone loss in rodents with periodontal disease and treated with resveratrol.

Study	Strain	Resveratrol Dose	ABL Evaluation Method	ABL		% Reduction	*p* Value
				Ligated with Resveratrol	Ligated without Resveratrol		
CORRÊA et al. (2018) [42] #	Wistar rats	10 mg/kg	Measurement of cementoenamel junction distance in methylene blue stain specimens	1.48 mm	1.61 mm	8.07%	0.0001
CORRÊA et al. (2018) [20] ¥	Wistar rats	10 mg/kg	Measurement of cementoenamel junction distance in methylene blue stain specimens	0.75 mm *	1.01 mm *	25.45%	<0.05
IKEDA et al. (2018) [44]	C57BL/6J wild-type mice	Single dose (intraperitoneal)	Measurement of cementoenamel junction distance in methylene blue stain specimens	65.00 µm *	165.00 µm *	60.60%	<0.01
CORRÊA et al. (2017) [41]	Wistar rats	10 mg/kg	Measurement of cementoenamel junction distance in methylene blue stain specimens	1.31 mm	1.41 mm	7.09%	<0.05
RIBEIRO et al. (2017) [40] ¥	Wistar rats	10 mg/kg	Measurement of cementoenamel junction distance in methylene blue stain specimens	0.12 mm *	0.15 mm *	20.00%	<0.05
BHATTARAI et al. (2016) [21]	Sprague–Dawley rats	5 mg/kg (subcutaneous)	Measurement of cementoenamel junction distance in hematoxylin and eosin stained slices.	55.00 µm *	130.00 µm *	57.69%	<0.05
			Measurement of bone mineral density in Micro CT specimens.	0.24 g/cm^3^ *	0.29 g/cm^3^ *	17.24%	
CHIN et al. (2016) [43]	Sprague–Dawley rats	25 mg/kg	Measurement of loss of periodontal bone-supporting ratio along the distal root surface junction in radiographic images of mandibles.	60.00% *	70.00%*	10.00%	<0.05
CIRANO et al. (2016) [39]	Wistar Rats	10 mg/kg	Don’t evaluated bone loss	-	-	-	-
ZHEN et al. (2015) [19]	C57BLKS/J-db/db mice	20 mg/kg	Measurement of cementoenamel junction distance in methylene blue stain specimens	0.19 mm *	0.31 mm *	−38.70%	<0.05
TAMAKI et al. (2014) [38]	Wistar rats	10 mg/kg	Measurement of distance from the cementoenamel junction to the alveolar bone crest in Micro CT specimens.	0.038 mm *	0.054 mm *	−29.63%	<0.001
CASATI et al. (2013) [37]	Wistar rats	10 mg/kg	Measurement of cementoenamel junction distance in methylene blue stain specimens.	0.61 mm *	0.71 mm *	−14.08%	<0.05

# Animals of both groups induced to periodontitis and arthritis. ¥ Animals of both groups submitted to cigarette smoke inhalation. * Values estimated by graphic data.

**Table 3:** An assessment of the risk of bias in the included studies.

STUDIES	A	B	C	D	E	F	G	H	I	J
CORRÊA et al. (2018) [42]	-	+	-	-	?	?	+	+	+	+
CORRÊA et al. (2018) [20]	+	+	-	?	?	?	+	?	+	+
IKEDA et al. (2018) [44]	+	?	-	?	-	-	-	?	+	+
CORRÊA et al. (2017) [41]	+	+	-	?	?	?	+	+	+	+
RIBEIRO et al. (2017) [40]	-	+	-	-	-	-	-	?	+	?
BHATTARAI et al. (2016) [21]	+	+	-	?	-	-	-	+	+	+
CHIN et al. (2016) [43]	-	-	-	?	-	-	-	?	?	?
CIRANO et al. (2016) [39]	-	+	-	-	+	?	+	?	+	+
ZHEN et al. (2015) [19]	+	+	-	?	-	-	-	?	+	+
TAMAKI et al. (2014) [38]	+	+	-	?	-	-	-	+	+	+
CASATI et al. (2013) [37]	+	+	-	?	?	?	+	+	+	+

A: Sequence generation. B: Baseline characteristics. C: Allocation concealment. D: Random housing. E: Blinding of participants and personnel. F: Random outcome assessment. G: Blinding of outcome assessment. H: Incomplete outcome data. I: Selective outcome reporting. J: Other bias. +: Yes (Low risk of bias). ?:Unclear. -: No (High risk of bias).

**Table 4:** The scores of the quality assessment according the Animal Research Reporting In Vivo Experiment (ARRIVE) guidelines of the included studies.

Studies		ARRIVE Items																			
	A	B	C	D	E	F	G	H	I	J	K	L	M	N	O	P	Q	R	S	T	Total
CORRÊA et al. (2018) [42]	1	1	1	1	2	2	2	2	2	1	1	2	2	1	2	2	2	1	2	2	32
CORRÊA et al. (2018) [20]	1	2	1	1	2	2	2	2	2	1	1	2	2	1	1	2	2	1	2	0	30
IKEDA et al. (2018) [44]	1	1	1	0	2	1	1	2	0	2	1	2	2	1	2	2	1	1	2	1	26
CORRÊA et al. (2017) [41]	1	2	1	1	2	2	1	2	2	1	1	2	2	1	1	2	2	1	2	2	31
RIBEIRO et al. (2017) [40]	1	1	1	1	2	1	2	2	1	1	1	2	2	1	1	2	2	1	2	2	29
BHATTARAI et al. (2016) [21]	1	1	1	1	2	1	1	2	1	1	1	2	2	1	2	2	2	1	2	2	29
CHIN et al. (2016) [43]	0	1	1	0	2	1	1	2	1	1	1	1	1	0	1	0	0	1	1	2	18
CIRANO et al. (2016) [39]	1	1	1	1	2	2	2	2	2	1	1	2	2	1	1	0	1	2	2	2	29
ZHEN et al. (2015) [19]	1	2	1	1	2	2	1	2	2	1	1	2	2	1	2	2	1	1	2	2	31
TAMAKI et al. (2014) [38]	1	2	1	1	2	1	1	2	2	2	1	2	2	1	2	2	1	1	2	2	31
CASATI et al. (2013) [37]	1	1	1	1	2	1	1	2	1	1	1	2	2	1	1	2	2	1	2	2	28
Category Score (Quality Obtained)	10	15	10	9	20	15	14	20	15	12	10	20	20	10	15	18	16	11	20	17	-
Maximum Score Expected (Quality Expected)	11	22	22	11	22	22	22	22	22	22	11	22	22	11	22	22	22	22	22	22	-
Ratio Quality Score/Maximum Score	0.91	0.68	0.50	0.82	1.0	0.72	0.68	1.0	0.72	0.59	1.0	0.95	0.95	0.91	0.72	0.82	0.72	0.54	0.95	0.86	-

A: title. B: abstract. C: introduction-background. D: introduction-objectives. E: methods-ethical statement. F: study design. G: experimental procedure. H: Experimental animals. I: housing and husbandry. J: sample size. K: allocation. L: experimental outcomes. M: statistics. N: results-baseline data. O: number analyzed. P: outcome, and estimation. Q: adverse events. R: discussion-interpretation/scientific implications. S: general applicability/relevance. T: funding. Total: represents total score obtained by each manuscript out of a maximum of 36 points.
